# CHALLENGES AND INSIGHTS: EVALUATING LIFE STORY WORK’S ROLE IN DEMENTIA CARE POST-PANDEMIC

**DOI:** 10.1093/geroni/igae098.4139

**Published:** 2024-12-31

**Authors:** Lacey DiFranco, Silvia Orsulic-Jeras, Sara Powers, Lisbeth Sanders

**Affiliations:** Benjamin Rose, Cleveland, Ohio, United States; Benjamin Rose, Cleveland, Ohio, United States; Benjamin Rose, Cleveland, Ohio, United States; LifeBio, Marysville, Ohio, United States

## Abstract

Decades of research have demonstrated that life story work can be an invaluable tool for residential care staff and individuals living with dementia. This study examines outcomes from 53 residential care staff (Mage=46.2 years, 92.5% female) implementing LifeBio Memory™ – an application that utilizes voice recorded interviews and artificial intelligence to automate production of resident story books and resources. In a pre-and post-intervention analysis utilizing resident proxy and staff evaluations, we observed an interesting distinction between the importance of person-centered care at the individual versus organizational level. We found a significant increase from baseline to follow-up among staff reporting they knew the resident’s life story [t(105) = -10.24, p <.001] and care preferences [t(106) = -8.91, p <.001] better after implementing the intervention. However, when asked about their feelings about and ability to provide person-centered care at their organization, staff reported a significant decrease from baseline (M = 39.70, SD = 5.54) to follow-up (M = 37.96, SD = 6.99) [t(52) = -1.42, p = 0.033]. More importantly, staff that reported higher agreeance to providing personalized care, also reported stronger organizational support at baseline (r = 0.427, p =.001) and follow-up (r = 0.606, p <.001). Additional feedback revealed organizational culture and direction for utilizing materials varied, and care facilities prioritized personal care over socialization and engagement when short-staffed. Discussion will focus on the role of organizational support for sustaining the use of life story materials in the provision of person-centered care within residential care settings.

